# Use of Stakeholder Feedback to Develop an App for Vestibular Rehabilitation–Input From Clinicians and Healthy Older Adults

**DOI:** 10.3389/fneur.2022.836571

**Published:** 2022-02-24

**Authors:** Linda J. DSilva, Karen M. Skop, Nathan T. Pickle, Katherine Marschner, Timothy P. Zehnbauer, Michael Rossi, Paulien E. Roos

**Affiliations:** ^1^Department of Physical Therapy, Rehabilitation Science, and Athletic Training, University of Kansas Medical Center, Kansas City, KS, United States; ^2^Physical Medicine and Rehabilitation Services, Department of Physical Therapy, James A. Haley Veterans' Hospital, Morsani College of Medicine, University of South Florida, School of Physical Therapy, Tampa, FL, United States; ^3^Biomedical and Data Sciences Division, CFD Research Corporation, Huntsville, AL, United States

**Keywords:** vestibular rehabilitation, physical therapy, vestibular ocular reflex, rehabilitation games, balance, rehabilitation application, dizziness

## Abstract

Close to half people over 60 years of age experience vestibular dysfunction. Although vestibular rehabilitation has been proven effective in reducing dizziness and falls in older adults, adherence to exercise programs is a major issue and reported to be below 50%. Therefore, this research aimed to develop an app with gaming elements to improve adherence to exercises that are part of vestibular rehabilitation, and to provide feedback to increase the accuracy during exercise performance. A clinician-informed design was used where five physical therapists were asked identical questions about the exercises they would like to see in the app, including their duration and frequency. Games were developed to train the vestibulo-ocular (VOR) reflex using VOR and gaze shifting exercises; and to train the vestibulo-spinal system using weight shifting and balance exercises. The games were designed to progress from simple to more complex visuals. The games were controlled by an Inertial Measurement Unit placed on the head or anterior waist. The app was tested on ten healthy females (69.1 ± 5.1 years) with no prior history of vestibular dysfunction or complaints of dizziness. Participants completed gaze stabilization and balance exercises using the app and provided feedback on the user interface, ease of use, usefulness and enjoyment using standardized questionnaires and changes they would like to see in the form of open-ended questions. In general, participants reported that they found the app easy to use, the user interface was friendly, and they enjoyed playing the games due to the graphics and colors. They reported that the feedback provided during the exercise session helped them recognize their mistakes and motivated them to do better. However, some elements of the app were frustrating due to incomplete instructions and inability to distinguish game objects due to insufficient contrast. Feedback received will be implemented in a revised version which will be trialed in older adults with dizziness due to vestibular hypofunction. We have demonstrated that the “Vestibular App^TM^” created for rehabilitation with gaming elements was found to be enjoyable, useful, and easy to use by healthy older adults. In the long term, the app may increase adherence to vestibular rehabilitation.

## Introduction

Vestibular hypofunction is a condition that results from damage to the vestibular organs in the inner ear and is associated with disabling symptoms including dizziness, vertigo, blurred vision, and postural instability. It is estimated that one third of US adults over 40 experience vestibular dysfunction and the prevalence increases sharply with age, with around half of those over age 60 experiencing vestibular dysfunction as documented by a balance test (1). It was found that those people who exhibit vestibular dysfunction symptoms (dizziness) are twelve times more likely to fall (1). Vestibular rehabilitation has been proven to be effective in reducing symptoms of dizziness and imbalance by providing exercise-focused interventions to promote adaptation and/or compensation for vestibular hypofunction (2–7). Clinical care is guided by best evidence, and in 2016 the Clinical Practice Guidelines (CPG) for vestibular hypofunction were created (8) and recently revised in 2021 (9). Based on CPG recommendations, vestibular-trained clinicians typically provide home exercise programs in written format as handouts and recommend patients perform the exercises consistently and accurately between 3 and 5 times a day, for a total of 15–20 min per day. However, adherence to the exercise program remains a major problem, with compliance rates below 50% (10). Many factors influence exercise adherence, however from a patient perspective the main factors that limit adherence to exercises are a lack of understanding of the exercises, fear of increasing symptoms during exercises, performing the exercises incorrectly, needing ongoing guidance and direction (10–13). Technology has made significant advances in the past two decades and can be harnessed to address some of these barriers.

Choi and colleagues found that persons who had suffered from a stroke and had resulting upper extremity impairments were more satisfied with a mobile game-based treatment vs. conventional treatment, they credit their success to the mobile game because it was specifically developed for their patients (14). For people with vestibular hypofunction, increased compliance and enjoyment was reported with the use of computer games, video games and using virtual reality (15–18). However, these games are not designed for vestibular rehabilitation at home, since they lack the specific head/body motions required to address gaze stability impairments, they are not customized based on each person's deficits, and cannot be advanced in visual or task complexity at a clinically relevant level as the person improves. Additionally, they do not provide patients with feedback to modify their performance, which currently is only possible when the person has direct supervision by a therapist.

Several studies have noted that ‘multifaceted' strategies are necessary to implement clinical practice guidelines and modify therapist behaviors (15–17). When one introduces technology to deliver interventions the clinician must learn new skills. Molding and colleagues found that having clinician feedback early in the development phase of technology can be helpful to adopt new clinical skills (18). Having clinician feedback and involvement early in the development phases of technology can help to shape the elements of technology that are essential in the clinic, keeping in mind clinician time and effort. It is also necessary to consider different types of clinics and perspectives of clinicians in different healthcare sectors. This is an important step to form an alliance between researchers, industry, and clinicians to maximize the probability that a novel technology will be adopted in clinical practice.

The aims of this study were to use a clinician-informed design to develop, implement and test an app to deliver a rehabilitation program congruent with the CPG for peripheral vestibular hypofunction. The goals of this study were: (1) to use structured interviews and clinician feedback to design an application, including defining the type of exercises, progression of exercises, and safety during exercise performance; and to (2) test a prototype application in healthy controls so that feedback from participants could be used to make iterative changes to create an app that could be used in people with vestibular hypofunction.

## Materials and Methods

### Clinician Feedback

To help shape the design of the virtual App-based technology for older adults with vestibular hypofunction, monthly focus group meetings were held with five physical therapists practicing in vestibular rehabilitation. All therapists hold a doctorate in physical therapy and have completed at least one course in vestibular rehabilitation. They work in a variety of settings including private practice, university hospital clinics, and Veterans' Affairs (VA) clinics. Years of experience range from 8 to 22 years, and they hold positions such as clinic owners, clinic directors, and lead vestibular therapists. Three therapists hold teaching positions. Each therapist participated in six one-hour sessions, that were setup at their convenience. The sessions were recorded and per session, identical questions were asked to all therapists involved. Responses were compared between therapists and with the CPG for peripheral vestibular hypofunction to identify gaps in practice.

Meetings focused on the following topics:

**Session 1–Identifying exercises to include**. The goal of this session was to identify and include all exercises that are required for older adults with vestibular hypofunction to perform their At-home rehabilitation session.

**Session 2–Defining requirements and instructions for exercises**. The purpose of this session was to define the instructions for each of the exercises that were included in the Vestibular Rehabilitation App^TM^. The information obtained was used to design each exercise to ensure that each game accurately represented the intent of the rehabilitation exercise.

**Session 3–Exercise progression**. The purpose of this session was to understand and describe patient progression in clinical practice. This provided information to shape the automated progression prompts in the app.

**Session 4–Techniques to improve adherence**. In this session, therapists discussed the barriers to adherence and techniques that are currently used to overcome them. Elements were designed and added to the app with the goal to improve compliance.

**Session 5–Patient education**. The purpose of this session was to identify the education provided by clinicians over the course of rehabilitation, such as the time it takes for recovery, the importance of walking and movement in the recovery process, among others. This session provided information to shape the educational elements in the app.

**Session 6–Safety measures**. The goal of this session was to define the safety features that would need to be included in the app for people to perform the exercises at home safely.

The specific questions that were asked in each session are in the Supplementary Material 1.

### Vestibular Rehabilitation Application

A mobile (smartphone or tablet) app containing games for the vestibular rehabilitation exercises was developed using Unity Pro (Unity Technologies) that could be used on an Android tablet. Games were controlled by an Inertial Measurement Unit [IMU; MetaMotionR (MBientlab)] that was connected to the tablet via Bluetooth. During gaze stability games, the IMU was placed on the forehead, and for the balance games, the sensor is secured with a waistband, just below the waist. The games developed were to train the vestibulo-ocular reflex (VOR) and gaze shifting or to train balance using weight shifting and single leg balance games. Artwork was developed specifically for the app. Care was taken to ensure that the artwork was appropriate for the target demographic (older adults) and has visual complexity that was tailored to patient severity.

#### Gaze Stability

##### VOR

**Figure 1:**
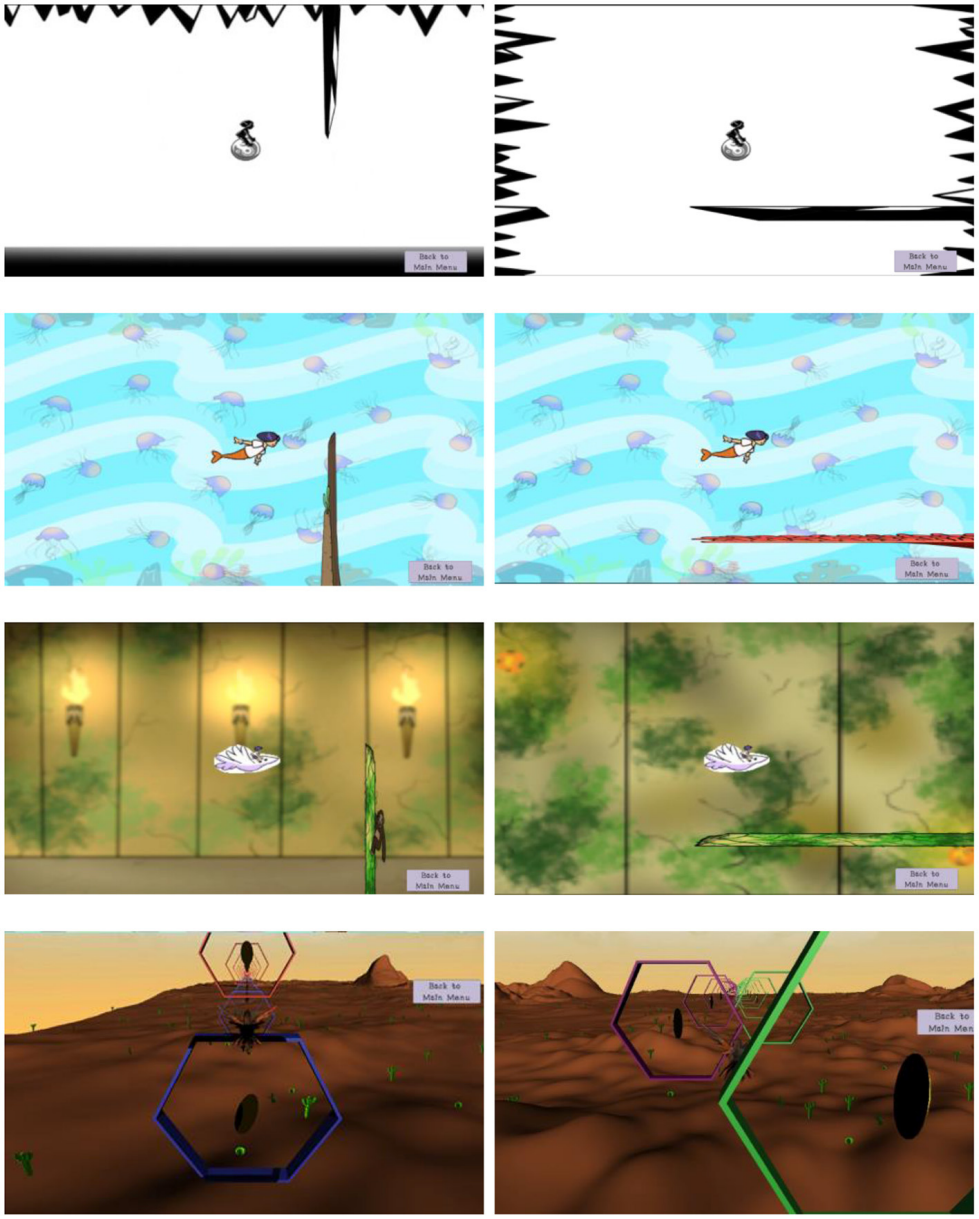
Four different levels of exercise for VOR in the pitch and yaw direction (left and right column respectively). The various levels of visual stimuli are tailored to the severity of symptoms due to vestibular dysfunction. Patients progress from simple backgrounds (top row), to a complex static background (2^nd^ row), a complex dynamic background (3^rd^ row–torches flicker), to a 3D background with texture and the character and background moving (bottom row). Note that although the background was static for the first two rows, the obstacles and character still moved over the screen.

The tablet was placed at arm's length for each individual so that they could reach the tablet easily to advance the games. A character on the screen was controlled by moving the head Up-Down (pitch) or Left-Right (yaw) to avoid obstacles. Points were awarded with each successful obstacle avoided. Four different levels of exercises for the VOR were designed which could be tailored to the rehabilitation needs at the clinicians' discretion (Figure 1). The first level had few distracting elements, and as the exercises progressed the background distractors increased. The final progression (level 4) was a 3D version of the VOR game, where the environment was dynamic with the character moving through a moving 3D scene. The app allowed the desired head range of motion and head angular velocity of the head motion to be changed. Changes in head range of motion were associated with the change in the amount of character movement relative to the head motion, so that the character was at the edge of the screen at the desired range of motion. Similarly, the head angular velocity setting of head motion controlled the speed at which the obstacles appeared on the screen. Speed was calculated using the range of motion and the desired angular velocity to set the amount of head rotations that needed to be made per second.

##### Gaze Shifting

**Figure 2:**
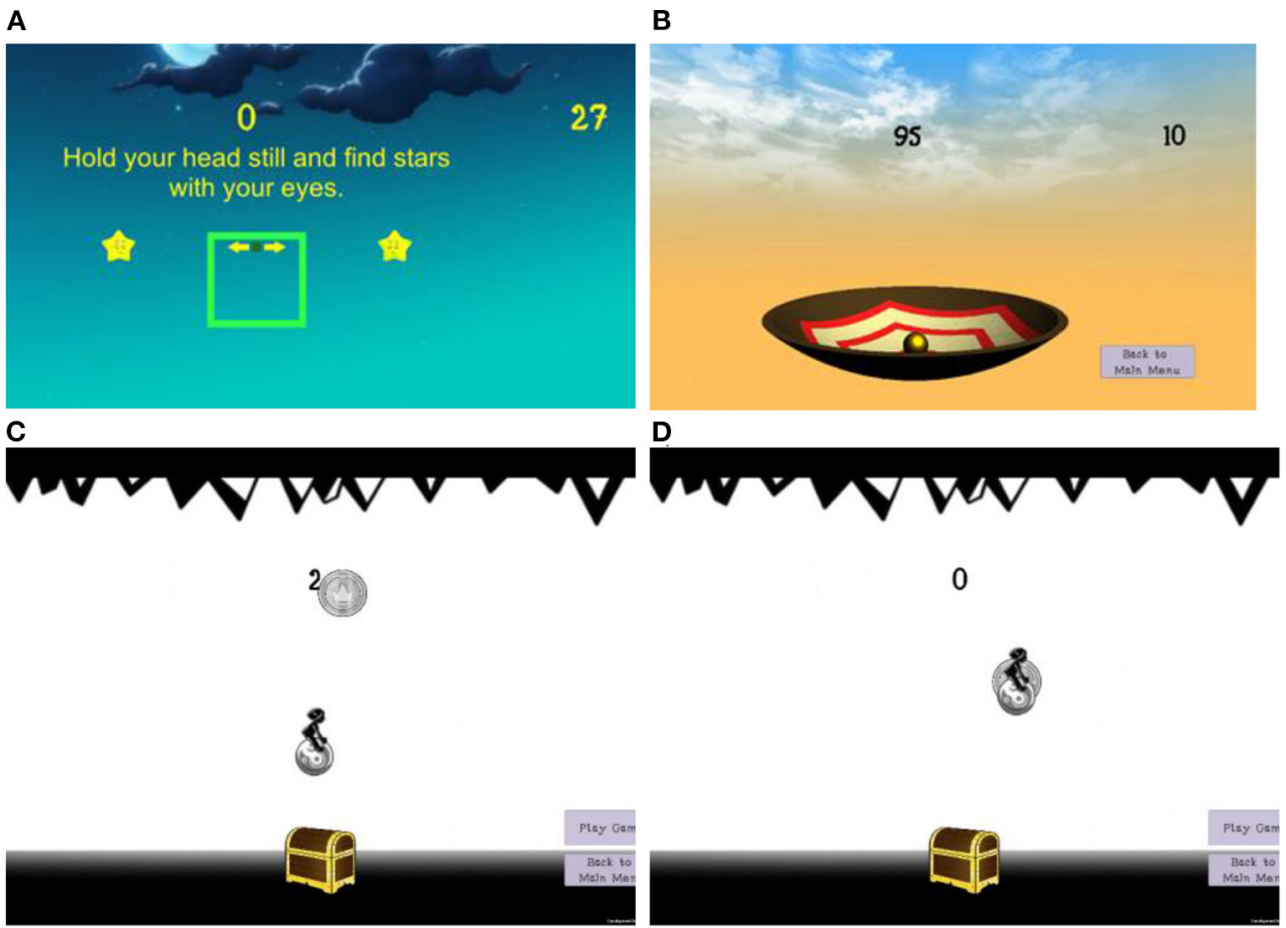
Screenshots of the different vestibular rehabilitation exercise games: **(A)** gaze shifting, **(B)** single leg balance, and **(C)** weight shift with the character moving toward the coin and **(D)** weight shift with the character returning the coin.

The tablet shows a 360° panoramic view of a night sky (Figure 2). With the tablet in hand, the participant has to move the tablet to find stars with their eyes only, without moving their head. On screen arrows point at the stars to guide the participant in the right direction. Once a star is aligned in the center of the screen, a message is provided with direction to move their head to align a box on the screen with the star. Success in this task is defined as a successful gaze shift exercise, with the eyes moving first followed by the head.

#### Balance Games

##### Single Leg Standing

A large bowl on the screen tilts anterior/posterior or laterally based on the movement of the IMU placed on the waist (Figure 2). The objective is to remain still and vertically aligned during single leg standing, keeping the ball in the center of the bowl (tilting causes the ball to roll away). Points are scored based on how close the ball is to the center of the bowl.

##### Weight Shifting

The person controls a character on the screen by shifting their body weight. Leaning forward moves the character up and leaning left/right moves the character horizontally. Instructions are given to move from the ankle and not from the hips or bend the knees. The goal is to collect the most coins and put these in a treasure chest to gain points (Figure 2). The coins appear at different locations on the screen so that the person must shift their weight in different directions.

### Human Subject Testing

The study was approved by the Institutional Review Board at the University of Kansas Medical Center (site PI-LD). The Vestibular Rehabilitation App^TM^ was tested with 10 adults, all participants signed informed consent before the study began. Inclusion criteria were: (1) Adults between 60 and 75 years of age; (2) Able to fluently read and speak English, (3) Able to give informed consent, (4) Able to get out of a chair independently. Exclusion criteria were as follows: (1) Pre-existing neurological disease, vestibular disease, or uncorrectable visual problem; (2) Lower extremity musculoskeletal injury or pain that would impede performing the balance exercises.

Human subject testing was performed in a laboratory setting at the University of Kansas Medical session, as a one-time session. During testing subjects were seated in a comfortable chair for the VOR exercises and standing for the balance exercises while facing a stand that the tablet was placed in with the tablet at eye level. The MetaMotionR IMU was placed on the subject's forehead for the gaze stability game and on the anterior waist for the balance games using hypoallergenic adhesive tape. The experimenter introduced the Vestibular Rehabilitation App^TM^ to the subject and only helped when needed. Subjects were asked to complete VOR games in both pitch and yaw exercise games at all four levels of visual complexity. Next, subjects completed the gaze shifting and balance games. Subjects played one set of each game and were given the option to rest between each game. After each game, participants were asked for feedback using a questionnaire that was developed specifically for the study. They rated the following questions using a 7-point Likert scale ranging from completely agree to completely disagree: (1) I enjoyed playing the game; (2) I understood what I was supposed to do in the game; and (3) It was easy for me to play the game. Followed by the following Open-Ended questions: (1) what I liked most about the game; (2) what I disliked most about the game. The questionnaire that was developed is included in the Supplementary Files.

After the participant has completed all exercises, they completed the following questionnaires.

1) Game Evaluation Questionnaire–The Game Evaluation Questionnaire was developed specifically for this study to evaluate each game separately (see Supplementary Material 2.1).
2) User Interface Questionnaire–The User Interface questionnaire (19) evaluates the overall reactions to the software. This rates several reactions on a scale of 0 to 9 (with 5 being a neutral answer).
3) Perceived Ease of Use Questionnaire–The perceived ease of use questionnaire by Davis (20) evaluates the ease of use of technology (see Supplementary Material 2.2).
4) Usefulness, Motivation, and Enjoyment Questionnaire–The usefulness, motivation and enjoyment questionnaire by Silveira et al. (21) was adapted to evaluate usefulness, motivation, and enjoyment when using the Vestibular App (see Supplementary Material 2.3).
5) Open Ended Feedback: At the end of the entire session, participants were asked five open-ended questions that were: (1) How challenging was the entire session? (2) What are the positive features of the app? (3) What did you not like about the app? (4) What changes would you like to see? (5) Do you see any barriers to using this app at home?

## Results

### Clinician Feedback

Outcomes of the structured interviews with the clinicians and how the feedback was incorporated in the Vestibular Rehabilitation App^TM^ are outlined below.

#### Session 1–Identifying Relevant Exercises

The main exercises prescribed were similar between therapists, with small variations in prescription of additional strengthening, habituation, and balance. Educational and motivational opportunities were also noted by each of the therapists, such as grounding exercises to aid in reduction of dizziness.

Based on this feedback, the exercises that were included in the Vestibular Rehabilitation App^TM^ were the VOR exercise in the pitch and yaw directions, the gaze shift exercise, weight shifting, and single leg balance exercise. Although some clinicians prescribed habituation exercises, these were not included in this preliminary version of the app. They will be included in future versions.

#### Session 2–Defining Requirements and Instructions for Exercises

Instructions for the VOR exercise were similar between therapists, with small differences such as focusing on a dot vs. a word on a card. Therapists agreed on the definition of correct exercise performance, however, differences were seen in exercise dosing. Questionnaires that were used by clinicians ranged from ABC (22), to DHI (23), to dizziness scales, to therapeutic outcomes questionnaires. All clinicians ask patients about activities that caused dizziness, however, they varied in the manner the information was used for further exercise prescription.

Based on this feedback, the Vestibular Rehabilitation App^TM^ was developed with flexibility to prescribe exercise duration and the support used (e.g., single vs. double leg stance, holding a chair) during balance exercises. A dizziness visual analog scale from 0 to 10 was added to the app to rate dizziness after the VOR exercises, as well as DHI and ABC questionnaires.

#### Session 3–Exercise Progression

There was consensus that patients could progress to performing exercises at a more difficult level after achieving the time and accuracy needed without increasing symptoms of dizziness excessively (as determined by a VAS Pre- and Post-exercise). Symptom resolution was factored into exercise progression where symptoms mostly resolved in 15–20 min after exercise performance. Typically, patients were progressed to a higher level after consistently accurate performance for 2 days.

Since the Vestibular Rehabilitation App^TM^ is intended to be a helpful tool for the clinician and not replace clinical expertise, an algorithm has been developed to alert the clinician when a patient may be ready to progress. This algorithm takes into account if the patient is doing the exercise correctly (consistently high scores and achieving prescribed motions) and without causing excessive dizziness (low reported change in dizziness after VOR exercises).

#### Session 4–Techniques to Improve Adherence

Therapists identified the hurdles to compliance to home exercises as: finding the time to do the exercises; not seeing immediate results; remembering to do the exercises; exercises increase symptoms of dizziness; exercises are boring; not sure if they are doing exercises correctly / uncomfortable doing exercises; patients think they should not do exercises when they are dizzy; no appropriate expectations of level of symptoms during exercises.

The following methods to improve adherence to exercise programs were identified: provide information on the importance of exercises and how to do them correctly; limit duration of exercise program (up to 15 min total); ask the patient how much time they could dedicate; recommend techniques to alleviate symptoms; Self-motivation and reward. All therapists reported that being responsive to patient's symptoms, identifying goals, and tracking progress toward these goals were key components for improving adherence.

Based on this feedback, automated reminders were incorporated in the Vestibular Rehabilitation App^TM^, providing reminders on the device where the app is installed. Exercises were turned into fun games. Rewarding elements, such as trophies and game scores were included to show progress made and to increase motivation. Future work will incorporate a storyline that is linked to patient progress toward their rehabilitation goals.

#### Session 5–Patient Education

Primary goals of education were to inform the patient on recovery trajectory, such as time to recovery, what to expect during rehabilitation and alternative strategies to help reduce their dizziness and improve balance.

Based on this feedback, educational messages in the Vestibular Rehabilitation App^TM^ are focused on positive messages that reinforce the importance of rehabilitation based on scientific evidence. The app has been programmed so that educational messages appear at random time points after completing a game. A link to patient resources is also included in the app, with background on vestibular disorders (24, 25), resources to get more active (26), and healthy living (27).

#### Session 6–Safety Measures

The safety measures identified when performing the exercises included: standing with the back to a corner and near a sturdy surface they could hold on to if needed; start in sitting before doing exercises in standing, clear the floor of clutter; make sure pets were out of the way; wear Non-skid shoes (no flip flops or slippers); stand on a firm surface (preferably no carpet); and not walking around while looking at the tablet.

Based on this feedback, safety instructions have been included in the Vestibular Rehabilitation App^TM^. Patients must acknowledge and agree to abide by the safety instructions to proceed to the exercise program.

### Application Testing

Subjects were 10 females aged 60–74 years, average age 69.1 ± 5.1 years. There were no adverse events noted during testing.

#### Results Game Evaluation Questionnaire

**Figure 3:**
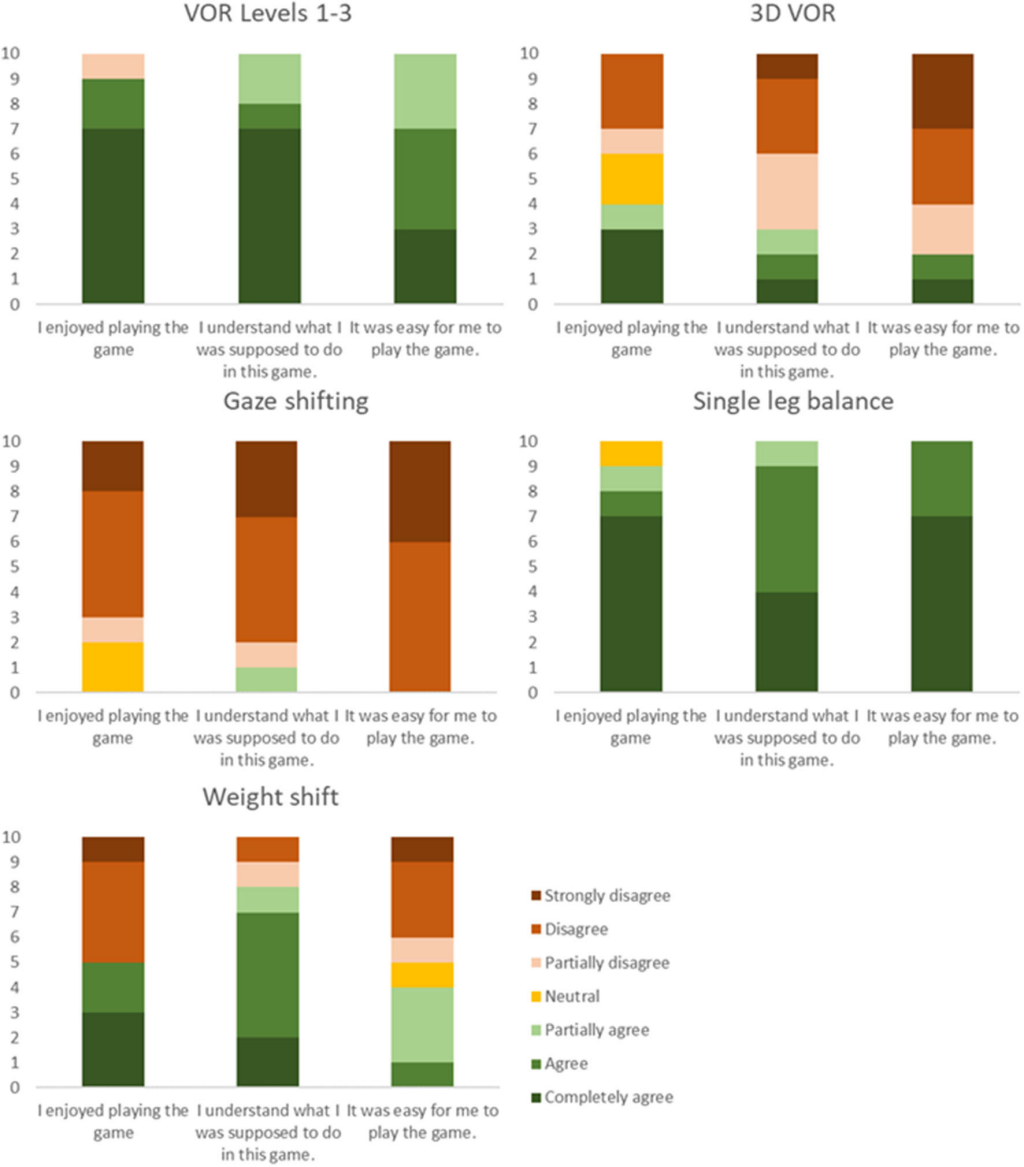
Ratings of the different games, indicating the number of subjects agreeing or disagreeing with the statements.

A mixed methods design was used to obtain detailed information about participant interaction with each game (gaze stability and balance) at each level. As well as elements of progression through levels 1–4. Subjects' response to questions regarding enjoyment, understanding game play and ease of play are shown in Figure 3. Responses to Open-Ended questions are summarized in the Supplementary Material 3.

In general, participants preferred the VOR Levels 1–3 and single leg balance games. Both games were easy to understand and were found enjoyable unlike the 3D VOR game which was difficult to understand. The gaze shifting game was the most difficult to understand and all agreed that they did not enjoy it. Although the instructions of the weight shifting game were clear, it was not easy, and participants had difficulty performing this exercise.

#### Results of the User Interface Questionnaire

**Figure 4:**
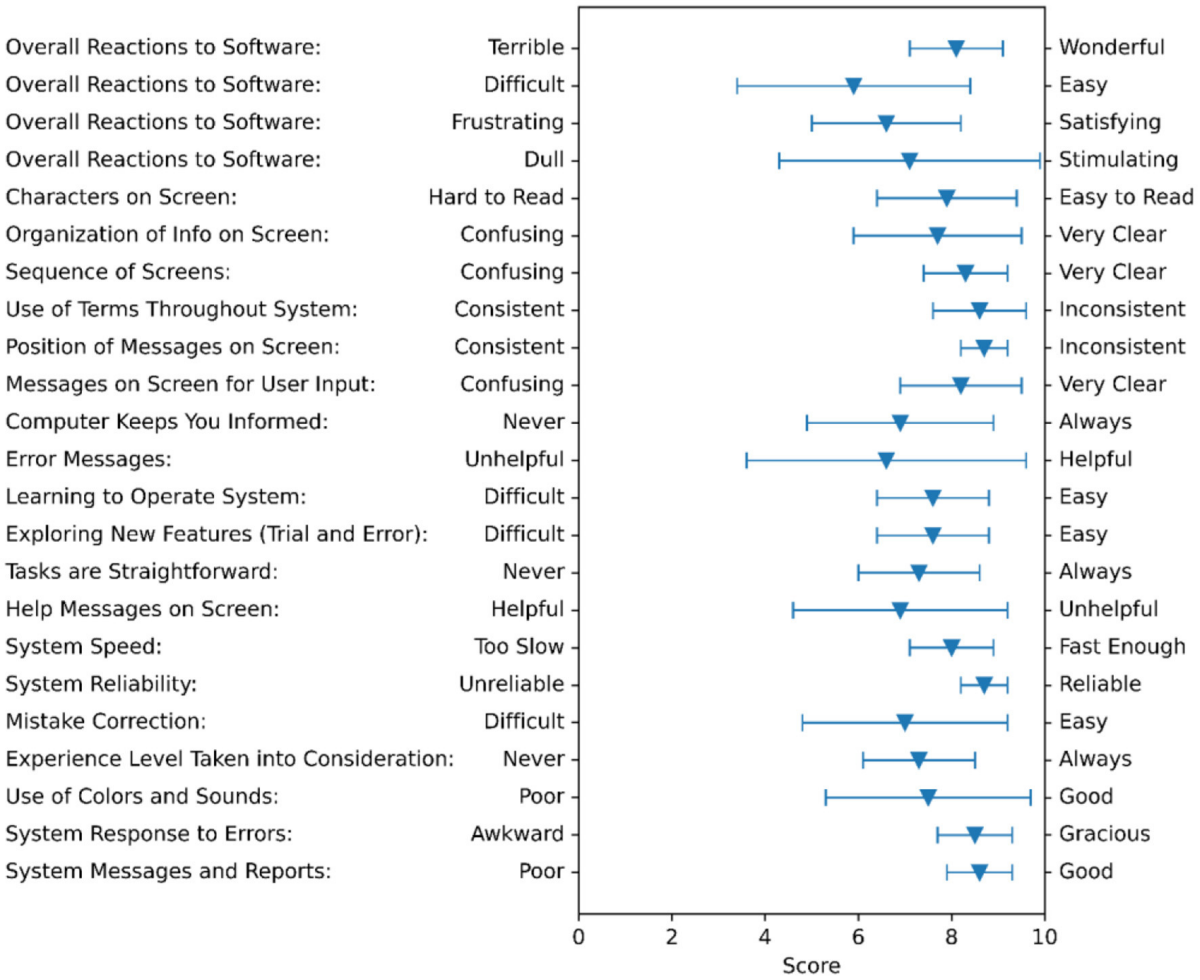
Feedback summary on the User Interface Questionnaire. The triangles show the averages of all subjects and the bars show the standard deviation.

Average ratings for each item are presented in Figure 4. Specific comments in response to each item can be found in the Supplementary Material 4.

#### Results of the Perceived Ease of Use Questionnaire

**Figure 5:**
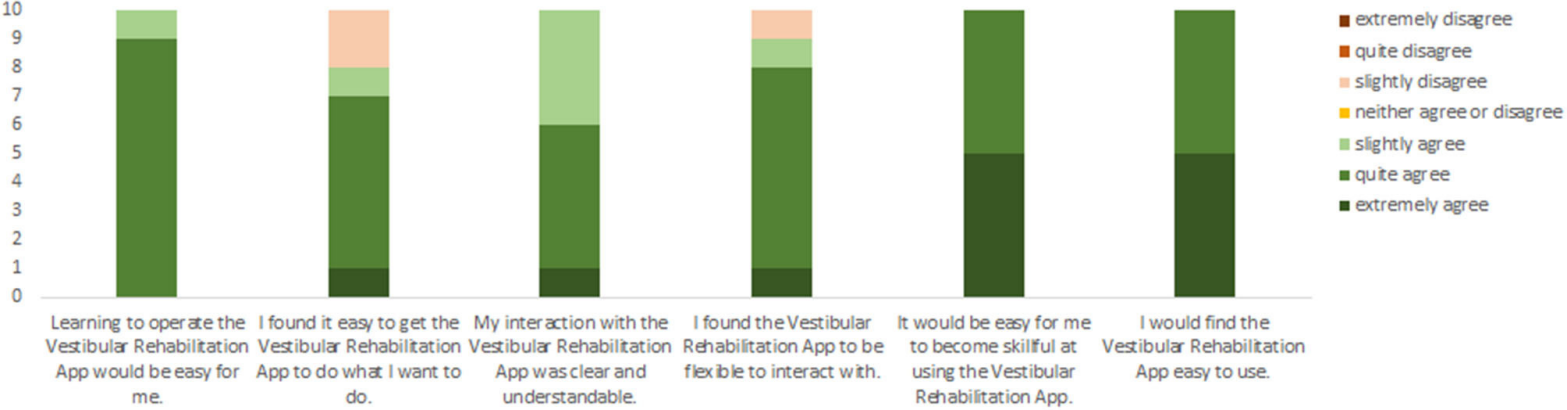
Rating for the six questions on the perceived ease of use questionnaire.

All participants indicated that learning to operate the “Vestibular Rehabilitation application” would be easy for them, their interaction with the app was clear and understandable, and it would be easy for them to become skillful at using the app (Figure 5). Eight out of the 10 subjects indicated that they found it easy to get the Vestibular Rehabilitation App^TM^ to do that they wanted it to do. Nine out of 10 people indicated that they found the Vestibular Rehabilitation App^TM^ to be flexible to interact with.

#### Results of the Usefulness, Motivation, and Enjoyment Questionnaire

**Figure 6:**
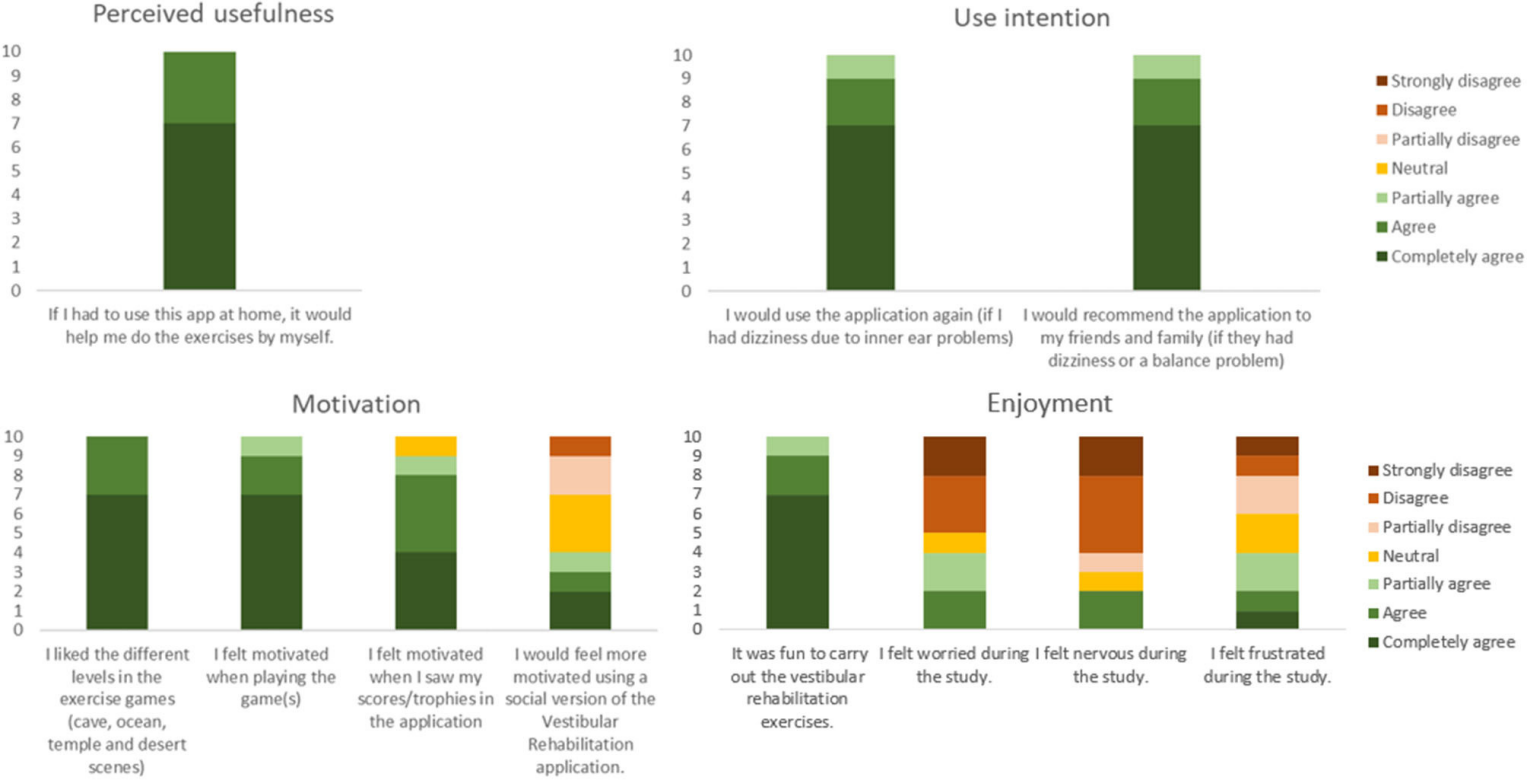
Overview of responses to the usefulness, motivation, and enjoyment questionnaire.

All participants agreed that if they had to use the app at home, it would help them do the exercises independently (Figure 6). They agreed that they would use the app and that they would recommend the app to their friends and family members with dizziness or a balance problem. All participants liked the different levels and felt motivated when playing the games. Nine participants felt motivated when they saw their scores or trophies in the application and 4 reported that they would feel more motivated using a social version of the application. All subjects responded that it was fun to carry out the vestibular rehabilitation exercises. Four subjects indicated that they felt worry and frustration, and only two subjects that they felt nervous while doing the exercises.

#### Open Ended Feedback

Five Open-Ended questions around the entire session concluded the session. Responses to the questions are reported below.

1) “How challenging was the session?” 5 subjects responded that the session was a good challenge, 2 responded that it was minimally challenging, 2 said that it was not challenging, but some games were difficult to understand, and 1 person responded it was challenging as she was not familiar with tablets or games. Most subjects indicated that some games were more challenging than others.
2) “What are the positive features of the app?” subjects responses included: visuals are great; active games; colorful and variety of games, enjoyed the competitiveness, liked the practice opportunity, instructions were straightforward, exercises got more challenging, using the sensor to control the games.
3) “What did you not like about the app?” were as follows: visibility and speed were too fast on some games, instructions on some games (3D VOR and gaze shifting) were confusing, frustration because they could not succeed in the games due to sensor not moving accurately.
4) “What changes would you like to see?” participants suggestions include: too hard to play the 3D VOR game, screen and character color similarities made it difficult to see the screen; Single-Leg balance was too easy, create varying degrees of difficulty; provide clearer instructions; improve graphics, identify goals or levels you try to reach, more positive reinforcement; toggle on/off audio–music or sound effects; provide feedback during the game about quality of movements (e.g., pause the game and give a reminder); explain the scores, as there was no reference to know if it was a good or a bad score.
5) “Do you see any barriers to using this app at home?” The main barriers identified were related to use of technology, the need to be comfortable using the tablet and sensor. Concerns about finances and having to buy a tablet and stand for the tablet, and placement of the tablet in a safe space in the home with adequate lighting and no glare.

## Discussion

The aims of this study were to use a Clinician-Informed design to develop, implement and test an app to deliver a rehabilitation program congruent with the CPG for peripheral vestibular hypofunction. Although technology has the potential to keep patients engaged by providing the exercises in a game format, with interesting storylines and graphics, exercises adapted into technology must be evidence based. The Clinical Practice Guidelines for Vestibular Hypofunction were used to guide exercise selection, prescription, and progression (8). Clinicians were included in the app design from the beginning and their feedback was considered at every stage to include elements in the app that addressed patient deficits, education, safety, and overall wellbeing.

Vestibular rehabilitation is an Exercise-Based approach that includes exercises to promote gaze stability, exercises to improve balance, habituation exercises to reduce symptoms, and walking for endurance. Of these, gaze stabilization exercises can be used either for adaptation or substitution of the vestibulo-ocular reflex depending on the severity of the hypofunction and if the involvement is unilateral or bilateral. Adaptation exercises require head movement while maintaining focus on a target, while substitution exercises use other strategies such as smooth pursuit eye movements to compensate for lost function. These exercises are effective in reducing symptoms of dizziness and fall risk after acute (28, 29) and chronic (6, 7) vestibular lesions as well as in older adults with complaints of dizziness without vestibular dysfunction (30). During structured interviews, all therapists involved in this study identified gaze stability and balance exercises as essential components of the rehabilitation program.

Poor adherence to vestibular rehabilitation exercises is a barrier that was reported by all clinicians involved in the study. To overcome the repetitive nature of the exercises and to engage patients during the program, the Vestibular Rehabilitation App^TM^ was designed with gaming elements, engaging graphics, and feedback on performance. We found that older adults with no prior experience with these games found the exercises engaging, the app interface easy to use, and felt motivated to do their best. However, results of our study show that to make the experience enjoyable, the games need to be clearly explained and the user interface needs to be intuitive. Additionally, the games that were too easy did not seem motivating but the games that were too difficult to perform or when the link between the sensor and the character on the screen was not precise, caused frustration. These factors such as simple instructions, intuitive user interface, and the right amount of challenge must be considered to keep patients engaged.

Participants were eager to know how they performed and reported that they appreciated the instant feedback to improve the accuracy of game performance but also wanted the terminal feedback to know what their total score was compared to the highest score possible. These are important factors that can increase motivation and thus adherence to the exercise program. Future iterations of the Vestibular Rehabilitation App^TM^ will include feedback strategies such as adding a story line, showing progress toward goals, and visually representing change over time. Artwork is important. Our findings show that artwork is a good way to get people to engage with the games. Several subjects remarked that they liked the graphics, though some also noted problems with contrast. When designing games for people who are sensitive to visual stimuli, it is important to consider color and graphics. For example, Whitney et al. (31) found that age and presence of dizziness can affect strategies to find objects in a virtual environment.

This study was to inform the design of the initial prototype version of the Vestibular Rehabilitation App^TM^ and to see where improvements were needed. Findings from this study will be considered in the development of future versions of the app. Future directions include creating a Web-Based prescription portal for therapists so that the number of exercises and their frequency can be easily prescribed. Additionally, accuracy of exercise performance and symptom severity with each exercise will be built into the portal and so that the results can be viewed easily in the form of graphs and tables, showing recovery trends over days, weeks, and months. Machine learning algorithms will be used to identify when mistakes in exercise performance are made so that the app can stop the exercise to provide feedback and ensure accurate performance. Validated questionnaires such as the Dizziness Handicap Inventory (23), and Activity-specific Balance Confidence scale (ABC) (22) will be incorporated into the app so that clinicians can track progress over time. This future development will get the app ready for testing on vestibular patients as well as clinical trials to demonstrate effectiveness of the app.

## Data Availability Statement

The raw data supporting the conclusions of this article will be made available by the authors, without undue reservation.

## Ethics Statement

The studies involving human participants were reviewed and approved by Institutional Review Board at the University of Kansas Medical Center. The participants provided their written informed consent to participate in this study.

## Author Contributions

PR and NP contributed to conception and design of the study. LD contributed to the design of the human subject testing and collected and summarized all data. PR analyzed, presented data, and wrote the first draft of the manuscript. LD and KS wrote sections of the manuscript. All authors contributed to manuscript revision, read, and approved the submitted version.

## Funding

Research reported in this publication was supported by the NIDCD of the National Institutes of Health (NIH) (R44DC017408; PI PR).

## Author Disclaimer

Content is solely the authors' responsibility and does not necessarily represent official views of the NIH, or represent official policy or position of the Department of Veteran Affairs or any other U.S. government agency.

## Conflict of Interest

NP, KM, TZ, and PR are employed by CFD Research Corporation and MR was employed by CFD Research Corporation at the time this research was conducted. The remaining authors declare that the research was conducted in the absence of any commercial or financial relationships that could be construed as a potential conflict of interest.

## Publisher's Note

All claims expressed in this article are solely those of the authors and do not necessarily represent those of their affiliated organizations, or those of the publisher, the editors and the reviewers. Any product that may be evaluated in this article, or claim that may be made by its manufacturer, is not guaranteed or endorsed by the publisher.
