# Supplementary material for: Use of Stakeholder Feedback to Develop an App for Vestibular Rehabilitation–Input From Clinicians and Healthy Older Adults
Source: Front Neurol. 2022 Feb 24;13:836571. doi: 10.3389/fneur.2022.836571 (PMC8907890; doi:10.3389/fneur.2022.836571)
Supplement: Supplementary file 1 [file Data_Sheet_1.docx]

Supplementary Material

**Table of Contents**

[1 Clinical Feedback Session Questions 2](#_Toc92357409)

[1.1 Session 1 – Identifying exercises to include 2](#_Toc92357410)

[1.2 Session 2 – Defining requirements and instructions for exercises. 2](#_Toc92357411)

[1.3 Session 3 – Exercise progression 3](#_Toc92357412)

[1.4 Session 4 – Techniques to improve adherence 3](#_Toc92357413)

[1.5 Session 5 – Patient education 3](#_Toc92357414)

[1.6 Session 6 – Safety measures 3](#_Toc92357415)

[2 Questionnaires 5](#_Toc92357416)

[2.1 Perceived Ease of use questionnaire 5](#_Toc92357417)

[2.2 Questionnaire to evaluate usefulness, motivation and enjoyment 7](#_Toc92357418)

[2.3 Game Evaluation Questionnaire 13](#_Toc92357419)

[3 Game Evaluation Questionnaire Open-Ended Responses 15](#_Toc92357420)

[4 Results Open-Ended Section of the User Interface Questionnaire 18](#_Toc92357421)

# Clinical Feedback Session Questions

## Session 1 – Identifying exercises to include

The questions asked in Session 1 to identify the exercises to include were as follows:

The purpose of Session 1 was to define the exercises to be included in the vestibular rehabilitation app for older adults. The goal is to include all exercises that are required for a typical older vestibular patient to perform their at-home rehabilitation.

The following questions were asked in this session:

1. How often do patients visit you in clinic?
2. How long is typical rehabilitation?
3. What exercises do you send patients home with?
4. How do you decide what exercises to prescribe?
5. What is typical frequency of exercises?
6. Can we categorize patients per condition?
7. What questionnaires/questions do you use?
8. How frequently do you take questionnaires/questions?

## Session 2 – Defining requirements and instructions for exercises.

The questions asked in Session 2 to define the requirements and instructions for the exercises were as follows:

The purpose of Session 2 was to define the requirements and instructions for each of the exercises to include in the vestibular rehabilitation app.

The following questions were asked in this session for each of the exercises identified in Session 1:

1. How do you instruct patient to perform this exercise?
2. How many repetitions / how often per day/week?
3. What are the requirements to perform the exercise correctly?
4. What parameters can you set/change per patient?
5. What would you like to know about the patient performing the exercise at home?
6. Any ideas/suggestions for games?

The exercises identified in session 1 were as follows:

1. 2 target VOR
2. Remember or imaginary targets
3. 3D VORx1 (optical flow)
4. Single leg balance
5. Weight shifting balance
6. Walking towards tablet while shaking head (with reservations because of safety concerns)

## Session 3 – Exercise progression

The purpose of Session 3 was to understand and describe patient progression in clinical practice.

The following questions were asked in this session:

1. How do you decide when a patient is ready to progress?
2. How do you decide when a patient has completed rehabilitation?
3. Any tests/assessments you use?
4. Is progression always forward?

## Session 4 – Techniques to improve adherence

The purpose of Session 4 was to understand hurdles to adherence and techniques to overcome these. This information will be used to add elements to the app that will further improve compliance.

The following questions were asked in this session:

1. What do you see as the main hurdle to adherence in this patient group?
2. What tricks do you use to improve adherence?
3. Do you use any self-management elements?

## Session 5 – Patient education

The purpose of Session 5 was to define what patient education is useful and if there is education that could be detrimental to the patient.

The following questions were asked in this session:

1. What patient education do you currently use?
2. Do you know of any good resources?
3. Is there any information/education that could be detrimental to patients?

## Session 6 – Safety measures

The purpose of Session 6 was to define the safety features that will be included in the app.

The following questions were asked in this session:

1. What safety measures do you use in clinic to prevent injury during exercises?
2. What safety precautions do you recommend to patients when they perform exercises at home?
3. What do you see as the main safety issues/risks for older patients when they perform vestibular exercises?
4. What suggestions do you have for safety precautions we could add to our app that normally cannot easily be implemented?
5. When should the app send safety alerts? What should trigger an alert?

# Questionnaires

## Game Evaluation Questionnaire

| I enjoyed playing the game | ○ Completely agree  ○ Agree  ○ Partially agree  ○ Neutral  ○ Partially disagree  ○ Disagree  ○ Strongly disagree |
| --- | --- |
| I understood what I was supposed to do in this game. | ○ Completely agree  ○ Agree  ○ Partially agree  ○ Neutral  ○ Partially disagree  ○ Disagree  ○ Strongly disagree |
| It was easy for me to play the game | ○ Completely agree  ○ Agree  ○ Partially agree  ○ Neutral  ○ Partially disagree  ○ Disagree  ○ Strongly disagree |
| What I liked most about the game |  |
| What I disliked most about the game |  |
| Any additional feedback? |  |

## Perceived Ease of use questionnaire

**Measurement Scale for Perceived Ease of Use**

This questionnaire is adapted from Davis et al.’s ^1^ questionnaire on ease of use of information technology. The only changes that were made to this questionnaire is changing the word “CHART-MASTER” to “Vestibular Rehabilitation App” and the word “job” to “rehabilitation”.

**Perceived Ease of Use**

| Learning to operate the Vestibular Rehabilitation App would be easy for me. | | | | | | | | |
| --- | --- | --- | --- | --- | --- | --- | --- | --- |
| likely |  |  |  |  |  |  |  | unlikely |
|  | extremely | quite | slightly | neither | slightly | quite | extremely |  |
| I find it easy to get the Vestibular Rehabilitation App to do what I want it to do. | | | | | | | | |
| likely |  |  |  |  |  |  |  | unlikely |
|  | extremely | quite | slightly | neither | slightly | quite | extremely |  |
| My interaction with the Vestibular Rehabilitation App was clear and understandable. | | | | | | | | |
| likely |  |  |  |  |  |  |  | unlikely |
|  | extremely | quite | slightly | neither | slightly | quite | extremely |  |
| I find the Vestibular Rehabilitation App to be flexible to interact with. | | | | | | | | |
| likely |  |  |  |  |  |  |  | unlikely |
|  | extremely | quite | slightly | neither | slightly | quite | extremely |  |
| It would be easy for me to become skillful at using the Vestibular Rehabilitation App. | | | | | | | | |
| likely |  |  |  |  |  |  |  | unlikely |
|  | extremely | quite | slightly | neither | slightly | quite | extremely |  |
| I would find the Vestibular Rehabilitation App easy to use. | | | | | | | | |
| likely |  |  |  |  |  |  |  | unlikely |
|  | extremely | quite | slightly | neither | slightly | quite | extremely |  |

## Questionnaire to evaluate usefulness, motivation and enjoyment

**Questionnaire to evaluate the Vestibular Rehabilitation Application**

This questionnaire is an adaptation of Silveira et al.’s (2013) questionnaire to evaluate the Active Lifestyle Application. The term “ActiveLifestyle” has been replaced with “Vestibular Rehabilitation App”, and “two week training plan” has been replaced with “rehabilitation session”. For question INT2, the following was added “(if they had a dizziness or balance problem)”. Questions M2-6 have been adapted to be relevant to the vestibular app. Motivational elements that are asked to be ranked have been adapted for the vestibular app. These are the only changes that were made to the original questionnaire.

1. **Details to be filled out by the interviewer**

Subject ID:

Date:

1. **Please answer the questions below taking into account your vestibular rehabilitation session using the Vestibular Rehabilitation Application.**
   1. **Perceived usefulness** (“the degree to which a person believes that using a particular system would enhance his or her performance”)

| PU1. If I had to use this app at home, it would help me do the exercises by myself | ○ Completely agree  ○ Agree  ○ Partially agree  ○ Neutral  ○ Partially disagree  ○ Disagree  ○ Strongly disagree |
| --- | --- |

- 1. **Use intention** (“a course of action that one intends to follow”)

| INT1. I would use the application again (if I were to need it due to vestibular issues, or just for fun) | ○ Completely agree  ○ Agree  ○ Partially agree  ○ Neutral  ○ Partially disagree  ○ Disagree  ○ Strongly disagree |
| --- | --- |
| INT2. I would recommend the application to my friends and family (if they had a dizziness or balance problem) | ○ Completely agree  ○ Agree  ○ Partially agree  ○ Neutral  ○ Partially disagree  ○ Disagree  ○ Strongly disagree |

- 1. **Motivation** (“providing with a reason to act in a certain way”)

| M1. I liked the different levels in the exercise games (cave, ocean, temple and dessert in the daring escape game) | ○ Completely agree  ○ Agree  ○ Partially agree  ○ Neutral  ○ Partially disagree  ○ Disagree  ○ Strongly disagree |
| --- | --- |
| M2. I felt motivated when playing the game(s) | ○ Completely agree  ○ Agree  ○ Partially agree  ○ Neutral  ○ Partially disagree  ○ Disagree  ○ Strongly disagree |
| M3. I felt motivated when I saw my scores/trophies in the application | ○ Completely agree  ○ Agree  ○ Partially agree  ○ Neutral  ○ Partially disagree  ○ Disagree  ○ Strongly disagree |
| M4. I would feel more motivated using a social version of the Vestibular Rehabilitation app, in which I could interact with other training partners | ○ Completely agree  ○ Agree  ○ Partially agree  ○ Neutral  ○ Partially disagree  ○ Disagree  ○ Strongly disagree |

Please, order the list of motivation strategies (for using the app) below using a numeric scale. For instance, 1 for the most motivating strategy, and 5 for the least motivating strategy.

|  | The scores in the individual games |
| --- | --- |
|  | The exercises being delivered as games |
|  | Being able to see my progress |
|  | The tips about the benefits of being active |
|  | The fact that my performance is being measured |

- 1. **Enjoyment**

| E1. It was fun to carry out the vestibular rehabilitation exercises. | ○ Completely agree  ○ Agree  ○ Partially agree  ○ Neutral  ○ Partially disagree  ○ Disagree  ○ Strongly disagree |
| --- | --- |
| E2. I felt worried during the study. | ○ Completely agree  ○ Agree  ○ Partially agree  ○ Neutral  ○ Partially disagree  ○ Disagree  ○ Strongly disagree |
| E3. I felt nervous during the study. | ○ Completely agree  ○ Agree  ○ Partially agree  ○ Neutral  ○ Partially disagree  ○ Disagree  ○ Strongly disagree |
| E4. I felt frustrated during the study. | ○ Completely agree  ○ Agree  ○ Partially agree  ○ Neutral  ○ Partially disagree  ○ Disagree  ○ Strongly disagree |

1. Silveira, P. *et al.* Tablet-Based Strength-Balance Training to Motivate and Improve Adherence to Exercise in Independently Living Older People: A Phase II Preclinical Exploratory Trial. *J. Med. Internet Res.* **15**, e159 (2013).

# Game Evaluation Questionnaire Open-Ended Responses

The table below summarizes the responses to the open-ended questions of the game evaluation questionnaire.

Table 1: Overview of the answers to the open-ended questions per game. The numbers in brackets after the answers indicates the number of people with similar responses.

| **Game** | **What I liked most about the game?** | **What I disliked most about the game?** | **Additional feedback** |
| --- | --- | --- | --- |
| **VOR** | It was straightforward  The fantasy, images, and color (3)  Challenge / skills needed (3)  Fun (2)  The different settings  It was engaging / motivating (2)  You have a goal | Hard to reset after mistake (2)  Making the child bump into things  Repetitive  Would like more feedback on whether doing it correctly  Neck might get tired  Did not dislike at all, just needed a little practice  More graphics, colors and details would have it kept more interesting  Wanted more variation and challenge as the levels went on  Did not like anything | Practices were helpful, took few minutes to get used to it (2)  Had difficulty turning head to the left.  Would like better graphics.  It was easy to learn even though I wasn't good at it  Difference between practice and actual games too big  Noticed the increasing challenge throughout the levels through the changing backgrounds  Fun  Enjoyed the changing graphics (2) |
| **VOR 3D** | Liked the graphics and the challenge  Would have liked it more than the 2D versions if the instructions were clear, since there was more "activity"  The challenge of the game  Was able to focus on coins  A little more challenging than 2D version, good speed, enjoyed the 3D aspects  Nothing, this was awesome | Speed  Rings were distracting, too many of them in view (3)  Game was complicated and instructions were not enough (5)  Visuals not easily distinguished (3)  Difficult to determine what to do (5), frustrating  Fast, a lot of stuff coming at you (3).  Did not feel like she got the coin, more positive feedback would be nice.  Struggled with the rhythm of it. | Sensor moves off to the side throughout the game making the participant lean further and further to their left  Coordination of head movement and character was difficult  Noticed herself trying to tilt her body and had to correct herself  Had difficulty with the depth perception. Hard to tell where the bird was in relation to the hoops |
| **2-target** | Concept was nice  Story line was good  Fun, colorful and cute (2)  Nice graphics  Nice to be able to hold the tablet  Did not dislike anything | Could not get it to do what I wanted it to do  Did not understand what I was supposed to do (6)  Instructions incomplete/unclear (6)  Did not work | Instructions disappeared too fast  Did not know whether to look at the stars in foreground or background  Add instructions on directions tablet can be moved  Maybe use a magnifying glass to first find the star and then the second square is a net to catch the star |
| **Single leg balance** | That I was successful / could do it (2)  Liked the game  It was easy  Unique and different from the others  Calming to watch the ball in the bowl  Challenged me (2)  Liked experimenting with different legs and with or without hands  Nothing I disliked (5) | It was not very challenging  Realizing how hard it was  It was a little boring  Did not totally understanding what the goal was  Not giving enough feedback, would have preferred the ball be more sensitive to movement | First screen of instructions doesn't make much sense  Add audio to the games for encouragement  Did not understand the scoring  Instructions indicate collecting coins to give a guard but there are no coins in the game |
| **Weight shift** | Fun / liked it (2)  Could see it would be good exercise to improve balance  Fun and challenging and unique, very mind-body game  Challenging | Lack of color difference between character and coin (2)  That I was unsuccessful (2)  Believed she had trouble because she was short  Did not understand what to do (2)  Did not like anything (2)  Felt there was a disconnect between her movements and the characters movements. (6) | Improve instructions  Improve the sync between sensor and movements (3)  Did not benefit from practice |

# Results Open-Ended Section of the User Interface Questionnaire

Responses to the open-ended sections of the User Interface Questionnaire (text box for comments and suggestions) were as follows:

“I think the more the exercises are done by the person would eliminate some of the frustration and stress”

“Using the software was challenging, sometimes found difficult in the games, but it was stimulating”

“Some (games) were easy to understand while a couple seemed confusing to me. The game with the stars was especially confusing retrieving the coin. The other games were fun and I could see a whole room of people competing against each other.”

“For me personally, the app started out easy and then progressed in difficulty.”

“More error messages (are) needed.”

“(I) Liked having the practice sessions available.”

“Practice was too slow compared to the speed of the game.”

“Perhaps increasing the challenge as you learn tasks, they became very easy. If I was using this app regularly I would like to be able to increase difficulty.”
